# The proportion of genes in a functional category is linked to mass-specific metabolic rate and lifespan

**DOI:** 10.1038/srep10008

**Published:** 2015-05-06

**Authors:** Kazuhiro Takemoto, Yuko Kawakami

**Affiliations:** 1 Department of Bioscience and Bioinformatics, Kyushu Institute of Technology, Iizuka, Fukuoka 820-8502, Japan

## Abstract

Metabolic rate and lifespan are important biological parameters that are studied in a wide range of research fields. They are known to correlate with body mass, but their association with gene (protein) functions is poorly understood. In this study, we collected data on the metabolic rate and lifespan of various organisms and investigated the relationship of these parameters with their genomes. We showed that the proportion of genes in a functional category, but not genome size, was correlated with mass-specific metabolic rate and maximal lifespan. In particular, the proportion of genes in oxic reactions (which occur in the presence of oxygen) was significantly associated with these two biological parameters. Additionally, we found that temperature, taxonomy, and mode-of-life traits had little effect on the observed associations. Our findings emphasize the importance of considering the biological functions of genes when investigating the relationships between genome, metabolic rate, and lifespan. Moreover, this provides further insights into these relationships, and may be useful for estimating metabolic rate and lifespan in individuals and the ecosystem using a combination of body mass measurements and genomic data.

Metabolic processes are essential for physiological functions and maintaining homeostasis in living organisms^1^,^2^. Therefore, understanding the factors that determine metabolic processes is an important topic of scientific inquiry, not only for researchers in the field of fundamental biology but also in ecology^3^,^4^ and medical research^5^. In particular, metabolic rate (especially respiratory rate) is an important physiological parameter for investigators in the fields of biology and ecology because it can be used to estimate, and therefore understand, energy metabolism, lifespan^6^,^7^, and animal space use^8^.

Previous studies have reported that metabolic rate strongly correlates with body mass; in particular, the relationship between metabolic rate *B* and body mass *M* approximately obeys a power law^4^,^9^: *B* *∝* *M*^*α*^ In general, α = 3/4 (such a scaling law is known as Kleiber’s law^4^,^6^,^9^,^10^); however, several previous studies suggest that Kleiber’s law may be not universal. For example, White and Seymour^10^ reported that metabolic rate is proportional to *M*^2/3^ in mammals, as predicted from the allometric scaling between body surface and body mass, when considering body temperature, digestive state, and phylogeny. Reich *et al.*^11^ observed a linear relationship between the rate of respiratory metabolism and body mass (i.e., *B* *∝* *M*) in plants. Similarly, the mean mass-specific metabolic rates observed across life’s major domains^12^ also implies that *B* *∝* *M*.

According to the rate of living theory, which predicts that animals with a greater rate of metabolism should die faster^6^, body mass is expected to be an important factor in determining lifespan. Indeed, several previous studies report an association between lifespan, body mass, and metabolic rate^6^,^7^,^9^,^13^, although other factors (e.g., ecological and phylogenetic) may also influence lifespan^14^.

Genomic information may also reflect metabolic rate because metabolic networks, which are expected to determine metabolic rate, are encoded in the genome^1^,^2^. In particular, metabolic rate is considered to be the consequence of many different biological reactions^15^ that involve proteins encoded by genes. However, several previous studies suggest that there is only a limited association between genomic information and metabolic rate/lifespan. For example, Kozłowski *et al.*^16^ and Starostová *et al.*^17^ reported that mass-specific metabolic rate is affected by cellular mass rather than by genome size or C-value (i.e., the amount of DNA in pg) in eyelid geckos. This may be because genome size has a weak correlation with cellular mass^13^. Although genome size is likely to positively correlate with lifespan in birds^18^ and fish^19^, some skepticism exists regarding the importance of genome size in fish (or homeotherms)^20^. In short, the association of genome size with metabolic rate and lifespan is controversial and is yet to be proven conclusively.

While previous studies imply that genomic information cannot be used to accurately estimate metabolic rate and lifespan, these studies themselves have limitations. In particular, they focused only on genome size or C-value as a measure of genomic information. The genome encodes several types of biological function, and the relationship between the number of functional genes and genome size differs according to functional category (e.g., defined by the Gene Ontology^21^) and species domain^22^,^23^. Therefore, in this study, we hypothesized that specific functional categories determine metabolic rate and lifespan rather than genome size. For example, genes related to oxic reactions or oxic metabolism^24^,^25^ (see also Methods), which are involved in processes that occur in the presence of oxygen, (hereinafter called *oxic genes*) may be useful for determining metabolic rate because they are expected to be involved in oxygen consumption.

Although the above previous studies mainly focus on C-value when considering genomic information, several new technologies and high-throughput methods have generated a large amount of genomic data, and such data are collected in several databases in recent years. Thus, investigators have become to be able to evaluate the relationship between genomic information and biological features such as metabolic rate and lifespan in more detail.

In general, such a relationship remains poorly understood. Therefore, here we investigated this relationship in more detail. Specifically, we collected data on metabolic rate and lifespan from published literature, selecting data from species for which a complete sequenced genome was available (see Methods). Using these data, we evaluated the usefulness of functional categories of genes for determining metabolic rate and lifespan.

## Results

### Genome size does not correlate with mass-specific metabolic rate

After the data collection and integration, we had obtained the genome, metabolic rate, and body mass data for 101 organisms, including 12 mammals, 9 protozoa, and 59 prokaryotes (see Methods and Supplementary Table S1).

A previous study^17^, Starostová *et al.* reported a limited effect of genome size, defined as the C-value, on mass-specific rate in eyelid geckos. However, this conclusion is limited to a specific family. Thus, we re-evaluated the correlation between genome size, defined as genome length (i.e., number of base pairs), and mass-specific metabolic rate (i.e., metabolic rate per body mass) in a wider range of species. Note that the definitions of genome size were different in our study and that of Starostová *et al.*^17^.

**Figure 1:**
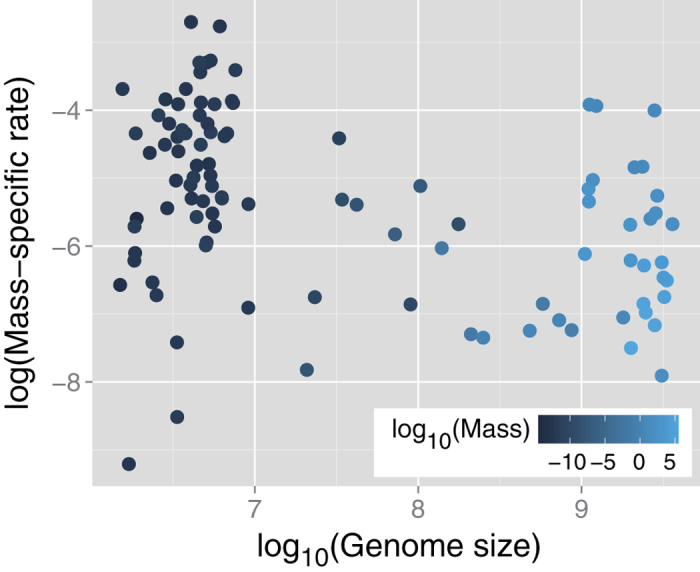
Scatter plot of mass-specific metabolic rate versus genome size Vertical and horizontal axes are on logarithmic and base-10 logarithmic scales, respectively. Symbol color indicates base-10 logarithmic body mass. The mass-specific rate negatively correlates with genome size (Spearman’s rank correlation coefficient *r*_*s*_ = –0.31, and *p* = 0.0013); however, it has no association with genome size when body mass is kept constant (partial rank correlation coefficient *r*_*s*_^*p*^ = –0.31, *p* = 0.0014).

We found a negative correlation between genome size and mass-specific metabolic rate (Spearman’s rank correlation coefficient *r*_*s*_ = –0.31, *p* = 0.0013) (Fig. 1). However, we observed a significant positive correlation between body mass and genome size (*r*_*s*_ = 0.83, *p* < 2.2 × 10^–16^). It remains possible that this observed relationship is a spurious correlation because it is well known that mass-specific metabolic rate shows a negative correlation with body mass^9^,^13^, despite some criticism^12^, In fact, in our dataset, we also found that there was a negative correlation between them (*r*_*s*_ = –0.42, *p* = 1.0 × 10^–5^). Especially, such a relationship between mass-specific rate *q* and body mass *M* is believed to follow a power law^9^,^13^: *q ∝ M*^*−β*^. Although *β* is expected to be 1/4 according to the Kleiber’s law^4^,^6^,^9^,^10^, a regression analysis (see Method) indicates that *β* is not zero but smaller than 1/4: *β* = 0.035 ± 0.008. This result is because of the fact that mean mass-specific metabolic rates are strikingly similar across life’s major domains^12^.

Additionally, we performed partial correlation analysis and found no correlation between genome size and mass-specific metabolic rate when body mass was kept constant (partial Spearman’s rank correlation coefficient *r*_*s*_^*p*^ = 0.076, *p* = 0.45). In contrast, a negative correlation was observed between mass-specific metabolic rate and body mass when genome size was kept constant (*r*_*s*_^*p*^ = –0.31, *p* = 0.0014).

This result implies a limited association between genome size and mass-specific metabolic rate, which provides evidential support for the findings of Starostová *et al.*^17^.

Temperature also affects metabolic rate^4^,^15^; therefore, we also considered temperature-corrected mass-specific metabolic rate (see Methods). In particular, we first evaluated the mass-specific metabolic rate at 25 °C (*q*_25_). Note that we only focused on approximately 80 organisms for which data on temperature was available (see Methods and Supplementary Table S1). We found no association between *q*_25_ and genome size both in a simple correlation analysis (*r*_*s*_ = –0.051, *p* = 0.66) and partial correlation analysis (*r*_*s*_^*p*^ = 0.16, *p* = 0.16). However, we need to consider a lower temperature because the organisms used in this study were primarily prokaryotes (see Supplementary Table S1) In particular, a lot of prokaryotes show optimal growth temperatures lower than 25 °C^26^. Thus, we next evaluated mass-specific metabolic rate at 10 °C (*q*_10_) (see Methods). Again, we confirmed no correlation between *q*_10_ and genome size in a simple correlation analysis (*r*_*s*_ = –0.13, *p* = 0.25) and partial correlation analysis (*r*_*s*_^*p*^ = 0.13, *p* = 0.23). These results suggest that a limited association between genome size and mass-specific metabolic rate can be also concluded when considering the effect of temperature on metabolic rate.

### Oxic genes are associated with mass-specific metabolic rate

As a simple measure of ‘functional category’, we considered the proportion of genes in a functional category (i.e., the number genes in the category divided by the total number of genes or proteins)^22^,^23^. This measure is well used to evaluate the predominance of genes in a functional category (i.e., function) between gene sets of different sizes (organisms with different genome sizes, in this case), in the context of functional enrichment analysis^27^. Note that the use of the number of genes in a functional category or the total number of nucleotides of genes in the category is not suitable for comparing the predominance between organisms with different genome sizes because genome size influences these parameters.

**Figure 2:**
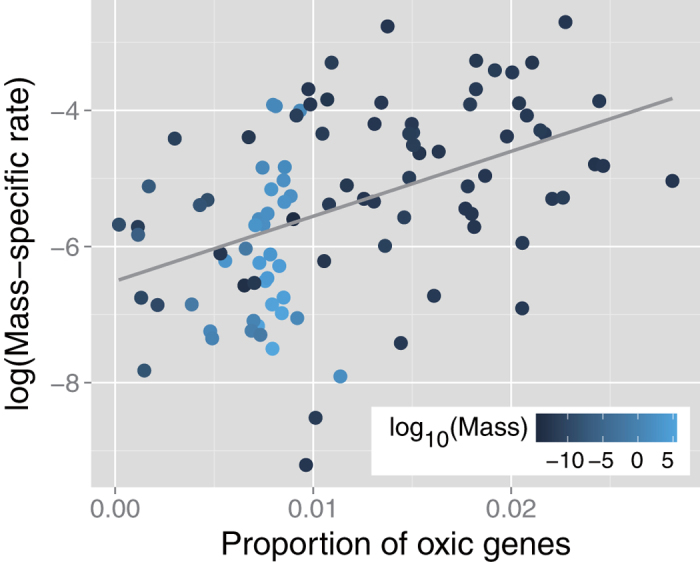
Scatter plot of the proportion of oxic genes versus mass-specific metabolic rate Vertical axis is on a logarithmic scale. The solid line is a linear regression fit to the data. Symbol color indicates base-10 logarithmic body mass. Both simple rank correlation analysis (*r*_*s*_ = 0.52, *p* = 2.0 × 10^–8^) and partial rank correlation analysis (*r*_*s*_^*p*^ = 0.38, *p* = 4.3 × 10^–5^), in which body mass is kept constant, indicate a positive association the proportion of oxic genes between the mass-specific rate.

We found a positive correlation between the proportion of oxic genes and mass-specific metabolic rate (*r*_*s*_ = 0.52, *p* = 2.0 × 10^–8^) (Fig. 2). Because the proportion of oxic genes was also associated with body mass (*r*_*s*_ = 0.55, *p* = 1.7 × 10^–9^), we performed a partial correlation analysis, correlating metabolic rate and the proportion of oxic genes while keeping body mass constant, and found a positive partial correlation (*r*_*s*_^*p*^ = 0.38, *p* = 4.3 × 10^–5^). Having removed the effect of the proportion of oxic genes, a negative trend was observed between metabolic rate and body mass; however, the partial correlation was not highly significant (*r*_*s*_^*p*^ = –0.19, *p* = 0.058). This result implies a limited effect of body size on mass-specific metabolic rate when considering the proportion of oxic genes in this dataset.

We also observed a positive correlation between the proportion of oxic genes and temperature-corrected mass-specific metabolic rate in both cases of 25 °C (*r*_*s*_ = 0.37, *p* = 7.2 × 10^–4^) and 10 °C (*r*_*s*_ = 0.37, *p* = 5.4 × 10^–4^). Furthermore, a positive correlation was found when the effect of body size was removed using partial correlation analysis in both cases of 25 °C (*r*_*s*_^*p*^ = 0.30, *p* = 0.0054) and 10 °C (*r*_*s*_^*p*^ = 0.27, *p* = 0.011). This tendency was also observed when focusing only on prokaryotes, in both cases 25 °C (*r*_*s*_^*p*^ = 0.28, *p* = 0.029) and 10 °C (*r*_*s*_^*p*^ = 0.28, *p* = 0.027). In contrast, there was no significant correlation between metabolic rate and body mass when the proportion of oxic genes was kept constant, in both cases of 25 °C (*r*_*s*_^*p*^ = –0.072, *p* = 0.53) and 10 °C (*r*_*s*_^*p*^ = –0.13, *p* = 0.22).

However, this general finding may be biased by the organisms examined in this study, which were primarily prokaryotes (see Supplementary Table S1). For example, when focusing only on higher organisms (i.e., mammals and birds), we found a negative correlation between mass-specific metabolic rate and body mass (*r*_*s*_^*p*^ = –0.96, *p* < 2.2 × 10^–16^) and a positive correlation between metabolic rate and the proportion of oxic genes (*r*_*s*_^*p*^ = 0.55, *p* = 0.0025). This finding suggests that there are some effect of taxonomy on the observed association between the proportion of oxic genes and mass-specific metabolic rate. However, in general, our results imply that oxic genes are associated with mass-specific metabolic rate, independent of body mass and temperature.

### The association between other functional categories and metabolic rate

Other functional categories are probably associated with mass-specific metabolic rate. To explore these, we evaluated Spearman’s rank correlations between the proportion of genes in functional categories, defined as a the Kyoto Encyclopedia of Genes and Genomes (KEGG) BRITE Functional Hierarchy^28^ (see Methods), and mass-specific metabolic rate. Additionally, we used partial correlation analysis to evaluate the association between functional categories and metabolic rate when body mass was kept constant.

Table 1 shows functional categories correlated with mass-specific metabolic rates (*p* < 0.05, using the partial rank correlation test). According to the effect sizes (i.e., correlation coefficients), oxic metabolism was the best estimator of mass-specific metabolic rate.

Along with oxic metabolism, mass-specific metabolic rate was associated with other functional categories. In particular, the observed association with cell motility is related to metabolic rate because cell motility corresponds to energy consumption in the context of flagellar motility and actin cytoskeleton dynamics. In addition, the association with energy metabolism, in which adenosine triphosphate (ATP) is generated, is directly linked to metabolic rate because the efficient generation of ATP requires oxygen in aerobes. The observed association with membrane transport may be linked to the fact that a large part of standard metabolic costs are spent preserving ionic gradients in cell membranes^16^,^29^,^30^. In addition to this, this result may also support the membrane pacemaker hypothesis of metabolism^31^,^32^, which proposes fatty acid composition of membrane determines metabolic rate.

However, the associations with xenobiotic biodegradation and metabolism and biosynthesis of other secondary metabolites are more difficult to interpret. They could be explained by the concentration of oxic reactions in such peripheral metabolic pathways^24^,^33^; thus, these associations may be artifacts caused by the primary observed association between oxic metabolism and mass-specific metabolic rate.

### Oxic metabolism, but not genome size, is associated with lifespan

According to the rate of living theory, metabolic rate is linked to lifespan. Consequently, we could hypothesize that gene function (specifically, the proportion of oxic genes) is also associated with lifespan. To test this hypothesis, we used data on the lifespan of 30 organisms, including 23 mammals and 7 birds, for which complete genome sequences were available (see Methods and Supplementary Table S2 for details). Based on our earlier observation of an association between the proportion of oxic genes and mass-specific metabolic rate in mammals and birds, we hypothesized that a negative correlation would exist between the proportion of oxic genes and lifespan in these animal groups.

**Figure 3:**
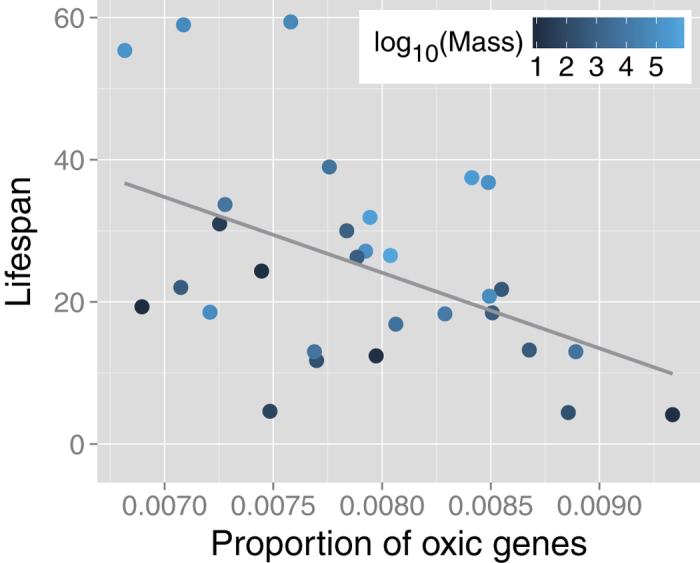
Scatter plot of the proportion of oxic genes versus maximal lifespan Symbol color indicates base-10 logarithmic body mass. The solid line is a linear regression fit to the data. Both simple rank correlation analysis (*r*_*s*_ = –0.42, *p* = 0.021) and partial rank correlation analysis (*r*_*s*_^*p*^ = –0.63, *p* = 1.8 × 10^–5^), in which body mass is kept constant, indicate a positive association the proportion of oxic genes between the mass-specific rate.

As expected, we found a negative correlation between the proportion of oxic genes and maximum lifespan (*r*_*s*_ = –0.42, *p* = 0.021) (Fig. 3). In several previous studies^6^,^7^,^14^, lifespan positively correlates with body mass. Here, we also observed a similar positive correlation (*r*_*s*_ = 0.58, *p* = 0.00074); thus, we performed a partial correlation to remove the effect of body mass. Independent of body mass, we still found a negative correlation between the proportion of oxic genes and lifespan (*r*_*s*_^*p*^ = –0.51, *p* = 0.0022). Note that this result does not directly indicate a limited effect of body mass on lifespan. We observed a positive correlation between body mass and lifespan when the proportion of oxic genes was kept constant (*r*_*s*_^*p*^ = –0.63, *p* = 1.8 × 10^–5^).

Ecological factors or mode-of-life traits can also affect lifespan. For example, Healy *et al.*^14^ reported that flight capability is the most important factor for longer lifespan in addition to body mass because volant species can more easily evade predators and unfavorable conditions. To test the effect of flight capability on the observed association between the proportion of oxic genes and lifespan, we separately evaluated a body mass-corrected partial correlation according to flight capability (volant/non-volant) (see Supplementary Table S2). We found negative correlations between the proportion of oxic genes and lifespan of 10 volant organisms (*r*_*s*_^*p*^ = –0.69, *p* = 0.012) and 20 non-volant organisms (*r*_*s*_^*p*^ = –0.48, *p* = 0.023). This result implies that the observed association between oxic metabolism and lifespan is independent of flight capability as a mode-of-life trait.

Using similar body mass-controlled correlation analysis, we found an association between lifespan and the sensory system including phototransduction and olfactory transduction (*r*_*s*_^*p*^ = –0.41, *p* = 0.020) and metabolism of cofactors and vitamins (*r*_*s*_^*p*^ = –0.38, *p* = 0.032). Several previous studies have shown that the sensory system is related to lifespan (reviewed in Refs.^34^,^35^). For example, Alcedo and Kenyon^36^ showed that gustatory neurons inhibit longevity. Our result is consistent with these previous studies. The latter association could be explained by the fact that oxic reactions or enzymes are known to frequently require iron, heme, and vitamins such as ascorbic acid as cofactors^33^. Similar to mass-specific metabolic rate, according to the effect size, the proportion of oxic genes was the best estimator of lifespan.

**Figure 4:**
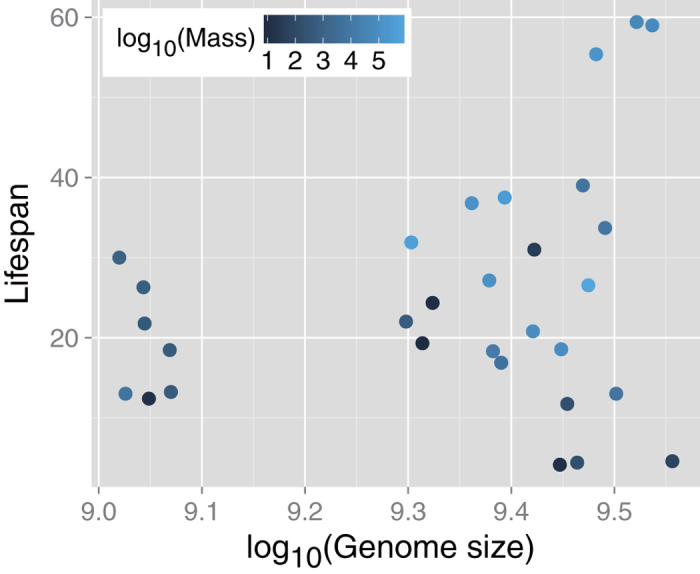
Limited correlation between genome size and maximal lifespan Horizontal axis is on a base-10 logarithmic scale. Symbol color indicates base-10 logarithmic body mass. A body mass-controlled (i.e., partial) rank correlation analysis indicated no association between genome size and lifespan (*r*_*s*_^*p*^ = 0.014, *p* = 0.94).

An association between genome size and lifespan is debatable because both positive^18^,^19^ and negative results^20^,^37^ have been reported. Thus, we re-evaluated this association using our dataset. Body mass-controlled partial rank correlation analysis indicated that there was no association between genome size and lifespan (*r*_*s*_^*p*^ = 0.014, *p* = 0.94) (Fig. 4).

## Discussion

Although metabolic rate and lifespan are well known to correlate with body mass^4^,^6^,^7^,^9^,^10^, their association with genomic information is unclear. Several previous studies^16^,^17^,^19^,^20^,^37^ have suggested that genome size or C-value correlates with metabolic rate and lifespan; however, such a correlation is controversial and has received criticism (e.g., Refs.^13^,^20^). Therefore, in this study, we performed a more detailed genomic analysis using several organisms with sequenced genomes, and demonstrated that the proportion of genes in functional categories was associated with mass-specific metabolic rate and lifespan. Conversely, we found that genome size was less informative for explaining metabolic rate and lifespan.

In particular, we found that the proportion of oxic genes was the best estimator of metabolic rate and lifespan because it is associated with both metabolic rate and lifespan. Because oxic genes are related to reactions that occur in the presence of oxygen, their association may reflect the importance of oxygen consumption in metabolic rate and lifespan. In addition, other functional categories such as cell mortality, membrane transport, and sensory systems were also associated with metabolic rate and lifespan, and the observed associations are is consistent with previous studies, as mentioned in Results.

However, more careful examinations are required to conclude what functional categories dominantly determine mass-specific metabolic rate and lifespan because we only performed correlation analyses. In particular, the result that multiple functional categories are linked to metabolic rate and lifespan may be because genes are overlapped among functional categories (i.e., multicollinearity). Ideally, we may need to perform a higher-level statistical analysis such as stepwise regression analyses. However, assumptions, such an analysis requires, (e.g., linearity and normality) are not satisfied in this study. In particular, several studies reported no universal scaling relationship in genome^22^,^23^. In addition to this, some outliers are observed. We were afraid that linear model-based analyses result in misleading conclusions. Thus, we could not considered linear model-based analyses. Although our results are consistent with biological understanding, they are still debatable. To avoid this problem, we need to consider a higher-level analysis and collection of biological data (details will be described below).

Our results do not entirely discount the possibility that genome size is associated to lifespan. Rather, they highlight the need for more detailed examination of relationships between genomic information and lifespan. In particular, we emphasize the importance of considering the biological functions encoded by the genome when exploring these relationships.

Although metabolic rate may be the result of many different biological reactions^15^, the specific reactions are not entirely clear. Our study suggests that the contribution of biological reactions to metabolic rate differs according to the reaction types. This finding is similar to a previous study, which reported that the body mass–metabolic rate relationship results from the sum of the influences of multiple contributors to metabolism and control^38^. Based on our results, oxic reactions are particularly useful for investigating metabolic rate and its association with environmental factors.

Metabolic rate is a primary focus in ecology because it is useful for estimating values such as animal space use^8^, lifespan^14^, and feeding rate^4^. In particular, the body mass–metabolic rate relationship has been actively investigated^4^,^6^,^7^,^9^,^10^. Our study suggests a possibility that genomic data are also used to estimate these ecological parameters. For example, machine learning methods (e.g., support vector machine and neural networks) may be useful. Support vector machine and neural networks are supervised learning models, and they are well used when predicting parameters from multidimensional data. Especially, these models are also applicable to nonlinear regression. For these reasons, machine learning methods have already been widely applied to predict biological features using genomic data in bioinformatics^39^. These methods require variables explaining (e.g., well correlated with) an objective variable (metabolic rate or lifespan, in this case). Thus, it is important to explore such explanatory variables. Indeed, we found that the proportions of genes in the several functional categories, including oxic metabolism, are associated with metabolic rate and lifespan. Using the patterns of functional gene contents (i.e., vectors of the proportions of these functional genes), machine learning methods may estimate metabolic rate and lifespan. Sequencing analyses are now beginning to be applied in ecology (e.g., in population ecology^40^ and for identification of species–species interactions^41^). This approach, in which genomic data are used to investigate the ecosystem, is known as *reverse ecology*^42^,^43^. We suggest that the findings in our study may be usefully applied in such research fields. However, more careful examinations are required to complete the prediction of ecological parameters using genomic data. This study merely reported an association between genomic data and ecological parameters, and it does not show the cause-effect relationship between them. In such a case, a prediction of ecological parameters from genomic data may be ineffective in real-world cases because of overfitting problem. To avoid this problem, we need to consider better methods of data analysis and data collection (details will be described below).

We acknowledge that the analysis we present here has some limitations. For example, the definition of functional categories is controvertible. Our conclusions are limited to the context of functional categories defined by the KEGG BRITE Functional Hierarchy^28^, and these definitions may be somewhat arbitrary (i.e., they depend on the database administrators). For a deeper understanding of the association between biological functions and metabolic rate and lifespan, better definition of functional categories will be required. For example, several studies focus on detecting functional modules (i.e., categories) using biological networks. This challenge is related to graph clustering or community detection of networks^44^,^45^,^46^. In addition, it is important to consider biological information such as reaction mechanisms, direction of reaction (i.e., reversible vs. irreversible), chemical structure of metabolites, and gene clusters. In this context, methods for finding biologically meaningful modules of biological networks based on gene clusters and chemical transformation patterns^47^,^48^ may be useful.

In contrast to some other functional categories, oxic genes or reactions^24^,^25^ have been defined by considering biological information using Scope^49^, a computational framework used to characterize the biosynthetic capability of a network when it is provided with certain external resources. Therefore, these genes/reactions may be more useful for explaining metabolic rate and lifespan. An extension of the method for detecting metabolic scope will also be important for exploring the association between genomic information and metabolic rate/lifespan.

The definition of the predominance of functional genes is controversial. In this study, we considered a simple measure: the proportion of genes in a functional category. It is still datable that this measure really reflects the predominance of functions. Ideally, we may need to consider the expression and activity of functional genes and metabolic enzymes using microarray and mass spectrometry^50^ because several previous studies^51^,^52^ have reported that activities of specific enzymes are linked to metabolic rate (i.e., oxygen consumption rate).

Here, we investigated only organisms for which genomes were complete and available; thus, our study was somewhat biased toward lower organisms such as prokaryotes. The observed correlation between genomic information and metabolic rate may, therefore, be more applicable to prokaryotes. However, we also observed a positive correlation between these factors in higher organisms such as mammals and birds. Additionally, we took steps in our methodology to reduce phylogenetic signals. Thus, we believe that the effect of taxonomy is unlikely to change our conclusion; however, we acknowledge that further careful examination will be required. The importance of phylogeny for evaluating associations between biological features is well known in terms of comparative phylogenetic analysis^53^,^54^,^55^. For example, several previous studies^10^,^12^ have reported that Kleiber’s law may be not observed when considering phylogenetic information. However, comparative phylogenetic analysis generally assumes a simple evolutionary model, which deems random Brownian-motion-like traits to be change on a phylogenetic tree with accurate branch lengths, and may, therefore, result in misleading conclusions. For instance, Griffith *et al.*^19^ pointed out the loss of statistical power that occurs when a dataset reduces in size because of phylogenetic corrections. Because our dataset contained only a few samples for higher organisms and, thus, falls into condition described by Griffith *et al.*, we did not consider comparative phylogenetic analysis.

The results of our study also depend on the quality of genome annotation. Furthermore, it is possible that our results are influenced by the percentage of functionally-unknown proteins in the study organisms. For metabolic networks, we already confirmed the difference in the fraction of functionally-unknown proteins between species categories in our previous study^25^. Thus, we believe that the quality of genome annotation would affect our conclusions; however, further research is required in this area. For example, metabolic networks are not fully understood. In particular, the existence of enzyme promiscuity^56^, which implies that enzymes can catalyze multiple reactions, act on more than one substrate, or exert a range of suppressions^57^, suggests the possibility of many hidden metabolic reactions, which may be related to metabolic robustness against changing environments^58^. Consideration of these hidden metabolic reactions will be important for designing metabolic pathways and for developing our understanding of metabolic evolution.

It will also be necessary to test the association between gene (protein) functions and metabolic rate and lifespan using additional organisms. Therefore, the continued sequencing of genomes from a wide range of organisms (including microorganisms such as extremophiles, mammals, fish, and insects) is obviously important. The development of high-throughput sequencing techniques will enable the collection of such data. For example, metagenomic techniques can now help to complete the sequencing of an organism’s genome.

Despite the limitations of our data analysis, our findings enhance the current understanding of the relationship between genomic information and the parameters metabolic rate and lifespan. Furthermore, they may be usefully applied in future research for estimating metabolic rate and lifespan using genomic data.

## Methods

### Data on metabolic rate, lifespan, and genome

We obtained data on metabolic rate and mass-specific metabolic rate from previously published literature^12^,^14^,^59^, which comprehensively reported the relationship between metabolic rate and body mass. We also extracted data on body mass and temperature from this literature.

The data on maximal lifespan were obtained from Healy *et al.*^14^. Additionally, data on body mass and species’ mode-of-life traits (i.e., volancy, fossoriality, foraging environment, and daily activity) were collected.

To prevent redundancy and reduce phylogenetic signals, we averaged the biological parameters (i.e., metabolic rate, body mass, and temperature) according to genus after unit conversion. The units of metabolic rate, mass-specific metabolic rate, body mass, and maximal lifespan were watts [W], watts per gram [W/g], grams [g], and years, respectively. We selected organisms for which genomes were available in the KEGG database^28^. We selected one species as a representative of a genus according to the year in which the species genome was first completely determined, and we used this genome for investigating the association between the biological parameters and the genomic information.

For investigating the association between metabolic rate and genomic information, we obtained data on 101 organisms including 12 mammals, 6 birds, 4 ectothermic vertebrates, 5 insects, 9 protozoa, and 59 prokaryotes (see Supplementary Table S1 for full details). In this dataset, the data on temperature were available for 84 organisms, including 12 mammals, 4 ectothermic vertebrate, 9 protozoa, and 59 prokaryotes.

For investigation of the association between lifespan and genomic information, we obtained data on 30 organisms, including 23 mammals and 7 birds (see Supplementary Table S2).

### Temperature-corrected mass-specific metabolic rate

To remove the effect of temperature^15^, mass-specific metabolic rate *q* obtained at different temperatures *T* [°C] was transformed to 25 °C: *q*_25_ = *q* × 10^–3^ × 2^(25 – *T*)/10^, according to a previous study^12^. In our study, we did not consider ectothermic vertebrates because these organisms do not live at a body temperatures of 25 °C^12^. Our dataset include only 4 (5% of the total) ectothermic vertebrates; thus, the exclusion of these organisms does not affect the conclusion. We can confirm that the similar conclusions can be obtained even if these ectothermic vertebrates using the dataset (Supplementary Table S1).

In addition to this, according to a previous study^15^, we also considered to transform *q* at observed at different temperature *T* [°C] to a given temperature *X* [°C]: *q*_*X*_ = *q* × exp[–*E*/*k* {1/(*X* + 273.15) −1/(*T* + 273.15)}], where *E* and *k* indicate an average activation energy [eV] for enzyme-catalyzed biochemical reactions and Boltzmann’ constant (i.e., *k* = 8.6173 × 10^–5^ [eV/K]). According to a previous study^15^, we considered *E* = 0.65 although *E* ranges between 0.4 and 0.8; but, we confirmed that similar conclusions can be obtained when *E* = 0.4 and 0.8.

### Identification of the oxic genes of each species

According to a database^24^ (prelude.bu.edu/O2/networks.html), we obtained an oxic reactions list based on Enzyme Commission (EC) numbers and metabolic reaction notations (i.e., reaction or ‘R’ numbers such as R00010) in the KEGG database^28^. Via the KEGG FTP site (ftp.bioinformatics.jp/kegg/xml/kgml/metabolic/organisms), on March 17, 2014 we downloaded XML files (version 0.7.1) containing the data on gene–reaction (i.e., gene identifier–R number) relationships of 111 organisms from the KEGG database. Based on this data, we defined oxic genes as genes associated with at least one oxic reaction.

### Functional categories of genes

In this study, we used the second level of KEGG BRITE Functional Hierarchy of the KEGG metabolic map (www.genome.jp/kegg-bin/get_htext?br08901.keg) for identifying functional categories of genes. We downloaded the data on functional category–gene identifier relationships of species S from the KEGG FTP site (ftp.bioinformatics.jp/kegg/brite/organisms/S/S00001.keg) on 17 March 2014, where S indicates the KEGG organism identifier (see Supplementary Tables S1 and S2).

In this study, we did not consider Gene Ontology^21^ as a definition of functional category because of fewer organisms whose GO annotations were completed, compared to KEGG BRITE Functional Hierarchy.

### Functional genome size

We computed the number of genes in a functional category and functional genome size (i.e., total number of nucleotides of genes in the functional category) (Supplementary Tables S1 and S2). For 111 organisms, we downloaded the nucleotide sequence data of species S from the KEGG FTP site (ftp.bioinformatics.jp/kegg/genes/organisms/S/) on 17 March 2014. On the basis of the functional category–gene identifier relationships, obtained as above, we calculated these two parameters.

Note that the KEGG FTP site was available only to paid subscribers as of 1 July 2011. Because the use of our data may be desirable to ensure reproducibility, our datasets are available upon request.

### Statistical tests

For measuring statistical dependence between parameters, we computed the Spearman’s rank correlation coefficient *r*_*s*_ (a non-parametric measure, which is relatively robust to outliers and can be used to analyze nonlinear relationships) and its associated *p* value using R version 3.1.1 (www.r-project.org).

To ensure that the results of the Spearman’s rank correlation analysis were robust, we also performed partial Spearman’s rank correlation analysis using R software. Specifically, we used the function *pcor*, available in the R package **ppcor** version 1.0.

To estimate the exponent of a power-law relationship, we performed a linear regression analysis using logarithmic values. In particular, we used the function *lm* in R software.

## Author Contributions

K. T. and Y. K. designed this study. K. T. and Y. K. collected the data, and K. T. performed data analysis. K. T. and Y. K. completed the manuscript and approved the final manuscript.

## Additional Information

**How to cite this article**: Takemoto, K. and Kawakami, Y. The proportion of genes in a functional category is linked to mass-specific metabolic rate and lifespan. *Sci. Rep.* **5**, 10008; doi: 10.1038/srep10008 (2015).

## Supplementary Material

Supplementary Table S1

Supplementary Table S2

## Figures and Tables

**Table 1:** Correlations between the proportion of genes in functional categories and mass-specific metabolic rate.

**Functional category**	***r***_***s***_	***r***_***s***_^***p***^
Oxic metabolism	0.52 (*p* = 2.0 × 10^–8^)	0.38 (*p* = 4.3 × 10^–5^)
Cell motility	0.33 (*p* = 7.3 × 10^–4^)	0.35 (*p* = 2.7 × 10^–4^)
Xenobiotics biodegradation and metabolism	0.42 (*p* = 9.9 × 10^–6^)	0.27 (*p* = 0.0069)
Biosynthesis of other secondary metabolites	0.45 (*p* = 2.9 × 10^–6^)	0.23 (*p* = 0.020)
Energy metabolism	0.43 (*p* = 8.7 × 10^–6^)	0.20 (*p* = 0.043)
Membrane transport	0.42 (*p* = 1.1 × 10^–5^)	0.20 (*p* = 0.048)

Spearman’s rank correlation coefficient (*r*_*s*_) and partial (i.e., body mass-controlled) rank correlation coefficients (*r*_*s*_^*p*^) are shown. The functional categories are displayed in descending order of *r*_*s*_^*p*^. Parenthetic values indicate the *p*-values.

## References

[b1] Takemoto K. Current understanding of the formation and adaptation of metabolic systems based on network theory. Metabolites 2, 429–457 (2012). 24957641 10.3390/metabo2030429 PMC3901219

[b2] Takemoto K. & Oosawa C. in Stat. Mach. Learn. Approaches Netw. Anal. 77–108 (2012). doi: 10.1002/9781118346990.ch3

[b3] Savage V. M. *et al.* The predominance of quarter-power scaling in biology. Funct. Ecol. 18, 257–282 (2004).

[b4] Brown J. H., Gillooly J. F., Allen A. P., Savage V. M. & West G. B. Toward a metabolic theory of ecology. Ecology 85, 1771–1789 (2004).

[b5] Milotti E., Vyshemirsky V., Sega M., Stella S. & Chignola R. Metabolic scaling in solid tumours. Sci. Rep. 3, 1938 (2013). 23727729 10.1038/srep01938 PMC3670262

[b6] Speakman J. R. Body size, energy metabolism and lifespan. J. Exp. Biol. 208, 1717–1730 (2005). 15855403 10.1242/jeb.01556

[b7] Niitepõld K. & Hanski I. A long life in the fast lane: positive association between peak metabolic rate and lifespan in a butterfly. J. Exp. Biol. 216, 1388–1397 (2013). 23264490 10.1242/jeb.080739

[b8] Jetz W., Carbone C., Fulford J. & Brown J. H. The scaling of animal space use. Science 306, 266–268 (2004). 15472074 10.1126/science.1102138

[b9] West G. B., Woodruff W. H. & Brown J. H. Allometric scaling of metabolic rate from molecules and mitochondria to cells and mammals. Proc. Natl. Acad. Sci. U. S. A. 99, 2473–2478 (2002). 11875197 10.1073/pnas.012579799 PMC128563

[b10] White C. R. & Seymour R. S. Mammalian basal metabolic rate is proportional to body mass^2/3^. Proc. Natl. Acad. Sci. U. S. A. 100, 4046–4049 (2003). 12637681 10.1073/pnas.0436428100 PMC153045

[b11] Reich P. B., Tjoelker M. G., Machado J.-L. & Oleksyn J. Universal scaling of respiratory metabolism, size and nitrogen in plants. Nature 439, 457–61 (2006). 16437113 10.1038/nature04282

[b12] Makarieva A. M. *et al.* Mean mass-specific metabolic rates are strikingly similar across life’s major domains: Evidence for life’s metabolic optimum. Proc. Natl. Acad. Sci. U. S. A. 105, 16994–16999 (2008). 18952839 10.1073/pnas.0802148105 PMC2572558

[b13] West G. B. & Brown J. H. The origin of allometric scaling laws in biology from genomes to ecosystems: towards a quantitative unifying theory of biological structure and organization. J. Exp. Biol. 208, 1575–92 (2005). 15855389 10.1242/jeb.01589

[b14] Healy K. *et al.* Ecology and mode-of-life explain lifespan variation in birds and mammals. Proc. R. Soc. B 281, 20140298 (2014). 10.1098/rspb.2014.0298 PMC4043093 24741018

[b15] Gillooly J. F., Brown J. H., West G. B., Savage V. M. & Charnov E. L. Effects of size and temperature on metabolic rate. Science 293, 2248–2251 (2001). 11567137 10.1126/science.1061967

[b16] Kozłowski J., Konarzewski M. & Gawelczyk A. T. Cell size as a link between noncoding DNA and metabolic rate scaling. Proc. Natl. Acad. Sci. U. S. A. 100, 14080–14085 (2003). 14615584 10.1073/pnas.2334605100 PMC283549

[b17] Starostová Z., Kubicka L., Konarzewski M., Kozłowski J. & Kratochvíl L. Cell size but not genome size affects scaling of metabolic rate in eyelid geckos. Am. Nat. 174, E100–E105 (2009). 19604072 10.1086/603610

[b18] Monaghan P. & Metcalfe N. B. Genome size and longevity. Trends Genet. 16, 331–332 (2000). 10904259 10.1016/s0168-9525(00)02051-5

[b19] Griffith O. L., Moodie G. E. E. & Civetta A. Genome size and longevity in fish. Exp. Gerontol. 38, 333–337 (2003). 12581799 10.1016/s0531-5565(02)00204-8

[b20] Gregory T. R. Genome size is not correlated positively with longevity in fishes (or homeotherms). Exp. Gerontol. 39, 859–860 (2004). 15130682 10.1016/j.exger.2004.01.015

[b21] Ashburner M. *et al.* Gene ontology: tool for the unification of biology. Nat. Genet. 25, 25–29 (2000). 10802651 10.1038/75556 PMC3037419

[b22] Molina N. & van Nimwegen E. Scaling laws in functional genome content across prokaryotic clades and lifestyles. Trends Genet. 25, 243–7 (2009). 19457568 10.1016/j.tig.2009.04.004

[b23] Koonin E. V. Are there laws of genome evolution? PLoS Comput. Biol. 7, e1002173 (2011). 21901087 10.1371/journal.pcbi.1002173 PMC3161903

[b24] Raymond J. & Segrè D. The effect of oxygen on biochemical networks and the evolution of complex life. Science 311, 1764–1767 (2006). 16556842 10.1126/science.1118439

[b25] Takemoto K. & Yoshitake I. Limited influence of oxygen on the evolution of chemical diversity in metabolic networks. Metabolites 3, 979–992 (2013). 24958261 10.3390/metabo3040979 PMC3937826

[b26] Thomas M. K., Kremer C. T., Klausmeier C. A. & Litchman E. A global pattern of thermal adaptation in marine phytoplankton. Science 338, 1085–1088 (2012). 23112294 10.1126/science.1224836

[b27] Huang D. W., Sherman B. T. & Lempicki R. A. Bioinformatics enrichment tools: Paths toward the comprehensive functional analysis of large gene lists. Nucleic Acids Res. 37, 1–13 (2009). 19033363 10.1093/nar/gkn923 PMC2615629

[b28] Kanehisa M. *et al.* Data, information, knowledge and principle: back to metabolism in KEGG. Nucleic Acids Res. 42, D199–D205 (2014). 24214961 10.1093/nar/gkt1076 PMC3965122

[b29] Porter R. K. & Brand M. D. Body mass dependence of H^+^ leak in mitochondria and its relevance to metabolic rate. Nature 362, 628–630 (1993). 8385274 10.1038/362628a0

[b30] Szarski H. Cell size and the concept of wasteful and frugal evolutionary strategies. J. Theor. Biol. 105, 201–209 (1983). 6656279 10.1016/s0022-5193(83)80002-2

[b31] Hulbert A. J. Life, death and membrane bilayers. J. Exp. Biol. 206, 2303–2311 (2003). 12796449 10.1242/jeb.00399

[b32] Rodriguez E. *et al.* Setting the pace of life: membrane composition of flight muscle varies with metabolic rate of hovering orchid bees. Proc. R. Soc. B Biol. Sci. 282, 20142232 (2015). 10.1098/rspb.2014.2232 PMC4344142 25652831

[b33] Jiang Y.-Y. *et al.* The impact of oxygen on metabolic evolution: a chemoinformatic investigation. PLoS Comput. Biol. 8, e1002426 (2012). 22438800 10.1371/journal.pcbi.1002426 PMC3305344

[b34] Kenyon C. A conserved regulatory system for aging. Cell 105, 165–168 (2001). 11336665 10.1016/s0092-8674(01)00306-3

[b35] Guarente L. & Kenyon C. Genetic pathways that regulate ageing in model organisms. Nature 408, 255–262 (2000). 11089983 10.1038/35041700

[b36] Alcedo J. & Kenyon C. Regulation of C. elegans longevity by specific gustatory and olfactory neurons. Neuron 41, 45–55 (2004). 14715134 10.1016/s0896-6273(03)00816-x

[b37] Morand S. & Ricklefs R. E. Genome size , longevity and development time in birds Genome size , longevity and development time. Trends Genet. 17, 567–568 (2001). 11642250 10.1016/s0168-9525(01)02414-3

[b38] Darveau C.-A., Suarez R. K., Andrews R. D. & Hochachka P. W. Allometric cascade as a unifying principle of body mass effects on metabolism. Nature 417, 166–170 (2002). 12000958 10.1038/417166a

[b39] Larrañaga P. *et al.* Machine learning in bioinformatics. Brief. Bioinform. 7, 86–112 (2006). 16761367 10.1093/bib/bbk007

[b40] Takahara T., Minamoto T., Yamanaka H., Doi H. & Kawabata Z. Estimation of fish biomass using environmental DNA. PLoS One 7, e35868 (2012). 22563411 10.1371/journal.pone.0035868 PMC3338542

[b41] Toju H., Guimarães P. R., Olesen J. M. & Thompson J. N. Assembly of complex plant–fungus networks. Nat. Commun. 5, 5273 (2014). 25327887 10.1038/ncomms6273 PMC4218951

[b42] Levy R. & Borenstein E. Reverse ecology: from systems to environments and back. Adv. Exp. Med. Biol. 751, 329–345 (2012). 22821465 10.1007/978-1-4614-3567-9_15

[b43] Klitgord N. & Segrè D. Ecosystems biology of microbial metabolism. Curr. Opin. Biotechnol. 22, 541–546 (2011). 21592777 10.1016/j.copbio.2011.04.018

[b44] Ahn Y.-Y., Bagrow J. P. & Lehmann S. Link communities reveal multiscale complexity in networks. Nature 466, 761–764 (2010). 20562860 10.1038/nature09182

[b45] Becker E., Robisson B., Chapple C. E., Guénoche A. & Brun C. Multifunctional proteins revealed by overlapping clustering in protein interaction network. Bioinformatics 28, 84–90 (2012). 22080466 10.1093/bioinformatics/btr621 PMC3244771

[b46] Fortunato S. Community detection in graphs. Phys. Rep. 486, 75–174 (2010).

[b47] Kanehisa M. Chemical and genomic evolution of enzyme-catalyzed reaction networks. FEBS Lett. 587, 2731–2737 (2013). 23816707 10.1016/j.febslet.2013.06.026

[b48] Muto A. *et al.* Modular architecture of metabolic pathways revealed by conserved sequences of reactions. J. Chem. Inf. Model. 53, 613–622 (2013). 23384306 10.1021/ci3005379 PMC3632090

[b49] Handorf T., Ebenhöh O. & Heinrich R. Expanding metabolic networks: scopes of compounds, robustness, and evolution. J. Mol. Evol. 61, 498–512 (2005). 16155745 10.1007/s00239-005-0027-1

[b50] De Rond T., Danielewicz M. & Northen T. High throughput screening of enzyme activity with mass spectrometry imaging. Curr. Opin. Biotechnol. 31, 1–9 (2015). 25129648 10.1016/j.copbio.2014.07.008

[b51] Thuesen E. V. & Childress J. J. Enzymatic activities and metabolic rates of pelagic chaetognaths: Lack of depth-related declines. Limnol. Oceanogr. 38, 935–948 (1993).

[b52] Thuesen E. V. & Childress J. J. Metabolic rates, enzyme activities and chemical compositions of some deep-sea pelagic worms, particularly Nectonemertes mirabilis (Nemertea; Hoplonemertinea) and Poeobius meseres (Annelida; Polychaeta). Deep Sea Res. Part I 40, 937–951 (1993).

[b53] Felsenstein J. Phylogenies and the comparative method. Am. Nat. 125, 1–15 (1985). 10.1086/703055 31094602

[b54] Garland T., Harvey P. H. & Ives A. R. Procedures for the analysis of comparative data using phylogenetically independent contrasts. Syst. Biol. 41, 18–32 (1992).

[b55] Garland T., Bennett A. F. & Rezende E. L. Phylogenetic approaches in comparative physiology. J. Exp. Biol. 208, 3015–3035 (2005). 16081601 10.1242/jeb.01745

[b56] Khersonsky O. & Tawfik D. S. Enzyme promiscuity: a mechanistic and evolutionary perspective. Annu. Rev. Biochem. 79, 471–505 (2010). 20235827 10.1146/annurev-biochem-030409-143718

[b57] Patrick W. M., Quandt E. M., Swartzlander D. B. & Matsumura I. Multicopy suppression underpins metabolic evolvability. Mol. Biol. Evol. 24, 2716–2722 (2007). 17884825 10.1093/molbev/msm204 PMC2678898

[b58] Nam H. *et al.* Network context and selection in the evolution to enzyme specificity. Science 337, 1101–4 (2012). 22936779 10.1126/science.1216861 PMC3536066

[b59] Kolokotrones T., Van Savage, Deeds E. J. & Fontana W. Curvature in metabolic scaling. Nature 464, 753–756 (2010). 20360740 10.1038/nature08920

